# The Book World of Medicine and Science

**Published:** 1906-02-03

**Authors:** 


### THE BOOK WORLD OF MEDICINE AND SCIENCE.

The Medical Epitome Series. Edited by Victor Cox Pederson, M.D., of New York. (London: Hodder and Stoughton, 1905. 16 vols. Price 4s. each net.)

This Epitome Series is an attempt to form a sort of pocket edition or Vade Mecum of Medicine. It comprises, in all, sixteen neatly bound, and, as medical books go, small volumes, the titles of which were given in The Hospital of January 6. The series is a very comprehensive one, ranging from "Inorganic Chemistry and Physics" to "Diseases of the Skin" and "Pediatrics." A number of questions have been arranged at the end of each chapter, embracing the subject matter. Dr. Pederson hopes the series will be both valuable and useful to the student and practitioner of medicine by reason of its comprehensiveness and moderate price, and of the brevity and clearness of each individual volume. In our opinion the series, taken as a whole, is disappointing, for this reason, that the principles and practice of medicine, surgery, and midwifery do not lend themselves easily to wholesale abbreviation. Although the series is a comprehensive one, it falls short of its object as regards both the medical student and the practitioner. The student will find it too cursory and too neglectful of detail, the general practitioner cannot use it much as a book of reference, and would find it more advantageous to purchase one or two of the many excellent up-to-date text books or monographs. The following volumes dealing with less appreciated but interesting branches of medicine are worthy of note because of their brevity and clearness: "Organic and Physiologic Chemistry," "Physics and Inorganic Chemistry," "Normal Histology," "Toxicology."

